# Skin-effect-mediated magnetoionic control of charge transport in thick layers

**DOI:** 10.1038/s41598-024-53970-9

**Published:** 2024-02-09

**Authors:** V. Barough, L. Jamilpanah, M. Zare, M. Ghanaatshoar, S. M. Mohseni

**Affiliations:** 1 Department of Physics, Shahid Beheshti University, Tehran, 1983969411 Iran; 2 Empa - Swiss Federal Laboratories for Materials Science and Technology, 8600 Dubendorf, Switzerland; 3 Laser and Plasma Research Institute, Shahid Beheshti University, Tehran, 1983969411 Iran

**Keywords:** Condensed-matter physics, Applied physics

## Abstract

In the rapidly developing area of magnetoionics (MI), which combines electrochemistry and magnetism, changes in the surface chemistry of magnetic materials in response to gate voltages cause dramatic modifications in the magnetic characteristics, resulting in low power-consuming charge transport tuning. Due to the surficial character, only magnetic thin films have been addressed for the MI effect’s role in controlling charge transfer. Here, we show how it can be used to regulate the transit of charges in bulk magnetic materials. This is accomplished by combining high-permeability magnetic materials with a high-frequency passing current, allowing the skin effect and the MI effect to control the magnetic materials’ impedance due to the impedance’s high sensitivity to magnetic permeability. Our in-situ impedance measurement and magneto-optical characterization show the role of redox reactions at the surface in controlling impedance in magnetic materials. This research paves the way for using the MI effect in high-permeability bulk magnetic materials.

## Introduction

Controlling the charge transport properties of magnetic materials has been the subject of research for many decades due to their high potential for application in memory and computing devices^1–5^. In magnetic materials, where the charge transport properties can be altered largely by controlling the magnetic properties, the means for controlling charge transport properties has been to apply an external magnetic field^6,7^, or to apply electric current^8^ to the material where magnetic torques mediate the switching of magnetic properties and therefore the charge transport properties. These methods include transport of charges in material and, therefore heating of the system^9–12^. This means higher energy consumption, which can result in technological problems with device functionalities. To overcome these problems, electric field control of magnetic properties has been followed through the magnetoionic (MI) effect in recent years^5^. In this method, an electric field is applied to an electrolyte (liquid or solid) in proximity to a magnetic material, and the chemical reactions at the surface of the magnetic material can induce the evolution of magnetic properties^13–20^. Some examples include tuning magnetization^2,9,21–23^, magnetic anisotropy^24–28^, and magnetization dynamics^24,29,30^. This method relies on redox reactions instead of the passing of charges in the material and therefore consumes less energy^17^. In addition, the nonvolatile nature of the chemical evolutions is another advantage that makes this method suitable for memory applications on corresponding devices^10,11^.

The limitation of the redox reactions to the surface of magnetic materials has limited the application of MI to only thin films. The most significant achievement of this study is the mediation of the skin effect, enabling the utilization of MI in bulk magnetic materials. In magnetic materials with high magnetic permeability, if an AC current passes through, the current will pass from the surface of the material in a skin depth $(\delta )$ that is dependent on the magnetic permeability $(\mu )$ of the material^6^. This effect, together with the surface-limited electrochemical evolutions in our sample, could result in the observation of an impedance change in the bulk magnetic material by ionic gating. In addition, our magneto-optical Kerr effect (MOKE) observations of the surface of the magnetic material show the evolution of the magnetic properties at the surface and therefore the impedance, which is permeability-dependent. According to the performed microscopic characterizations, the change in the magnetic properties at the surface is due to the oxidation of the sample at the surface. During the oxidation process at the surface of the sample, irreversible modifications have also occurred in addition to reversible manipulations, which will be investigated in detail in the next sections.

## Results and discussion

**Figure 1:**
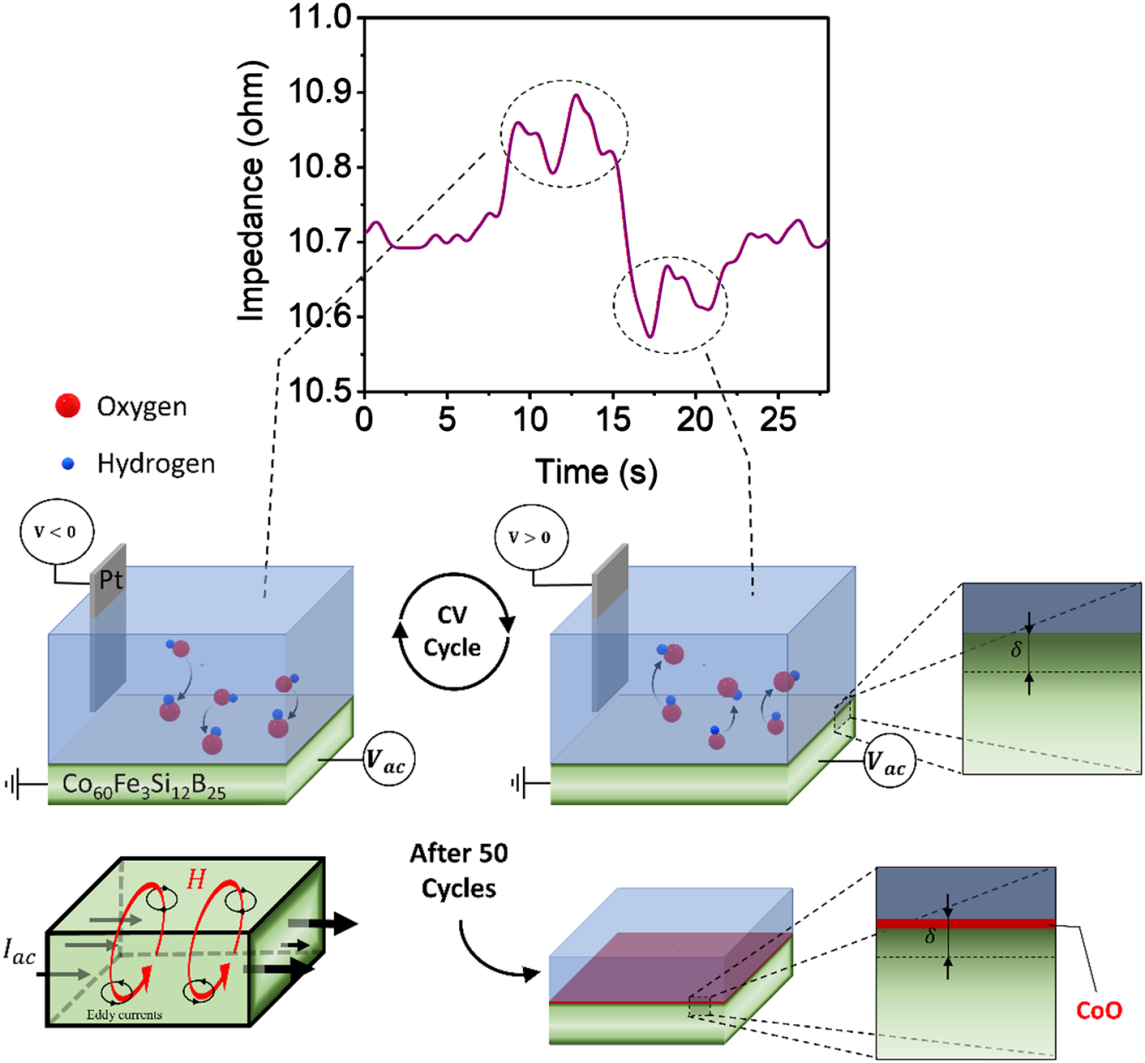
The schematic illustrates in-situ electrochemical and impedance measurements, at 8 MHz. Oxidation and reduction reactions cause impedance to increase and decrease, respectively. After 50 cycles of cyclic voltammetry (CV) test, the surface of the sample partially oxidizes, which also affects the impedance.

The general concept under examination is depicted in Fig. 1. To test the in-situ impedance of cobalt-based amorphous ribbons (Co_60_Fe_3_Si_12_B_25_), a particular setup has been developed. Eddy currents cause the core current in a magnetic material to be diminished when an alternating current is supplied. On the other hand, there is a concentration of current at the surface due to an increase in the current density at the material's surface. Actually, the impedance and the cyclic voltammetry (CV) test were performed at the same time. When the electrochemical CV test is conducted at the sample surface, two types of manipulation could be achieved in the sample structure. Firstly, the hydroxide agents in each CV cycle partially bonded and detached the sample's surface in the 1 molar potassium hydroxide electrolyte, respectively, by swiping voltage negatively and positively. The surface underwent two oxidations and reductions upon voltage increase and decrease, resulting in an increase and decrease in the impedance with each oxidation and reduction. These electrochemical reactions can be identified by fully reversibly monitoring the impedance at a frequency of 8 MHz. The next section will go deeper into the examination of this observation of reversible alterations at the sample surface. Secondly, portions of the sample surface remained oxidized after each CV cycle, which could have an irreversible effect on the impedance. As a result, it is reasonable to assume that the sample's impedance would gradually increase due to the metal oxide's poorer conductivity and lower permeability than that of metallic cobalt and iron. Additionally, the sample attained a saturated level of oxidation, and the impedance reached a stable quantity where only the reversible phenomena could be identified after completing multiple CV tests (around 50 cycles). In this study, we will investigate both reversible and irreversible manipulation of the magnetic properties of the soft ferromagnetic cobalt-based amorphous ribbon. It is noteworthy that the impedance curve has been smoothed in order to improve peak clarity; Figure S2 of the supporting information provides the raw data as well as the specifics of the smoothing procedure.

**Figure 2:**
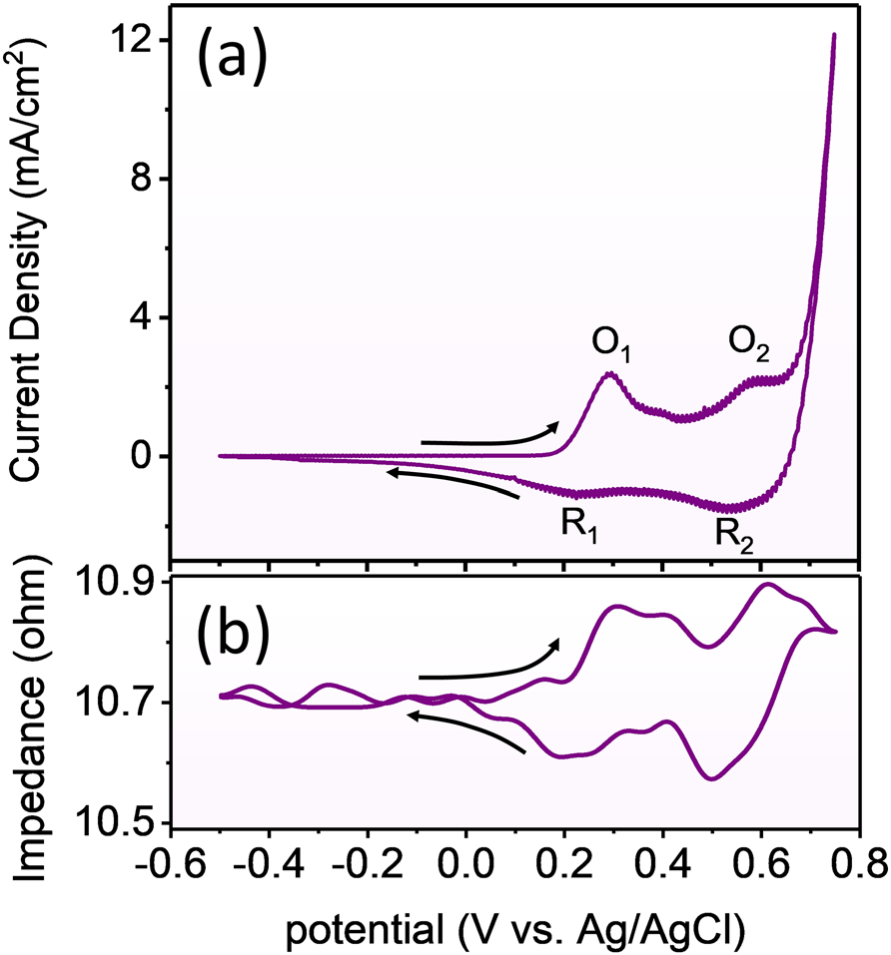
(**a**) Electrochemical current as a function of potential for cobalt-based amorphous ribbon in 1M KOH (pH = 14). (**b**) Variation of impedance at 8 MHz that was synchronized with the electrochemical reaction. In this diagram, impedance changes as a function of potential.

In order to investigate the reversible manipulation of the magnetic properties of cobalt-based amorphous ribbon (Co_60_Fe_3_Si_12_B_25_), the impedance measurement and CV test have been performed simultaneously. Figure 2a presents the electrochemical current density as a function of potential (V) for the sample. The potential starts at − 0.5 V and is swept towards the positive potential. The main characteristics that can be seen in the CV diagram are four peaks, which are named (O1) at 0.29 V, (R1) at 0.23 V, (O2) at 0.59 V, and (R2) at 0.54 V, respectively. Peaks O1 and R1 correspond to the transition between Co^2+^ and Co^3+^, and peaks O2 and R2 correspond to the transition between Co^3+^ and Co^4+^^31^ (a sharp increase in current density at potentials above 0.67 V is related to the oxygen evolution reaction (OER)). Figure 2b shows the impedance diagram measured simultaneously with the electrochemical reaction at the frequency of 8 MHz. This diagram shows the impedance changes as a function of potential. By sweeping the voltage of the electrochemical measurement from − 5 V, the impedance remains unchanged at the value of 10.7 Ω until an oxidation reaction occurs. Then it increases by 0.2 Ω and decreases by 0.1 Ω simultaneously with each oxidation and reduction reaction, respectively. Furthermore, no rise in impedance is observed when the voltage is increased further to the OER reaction range, suggesting that the redox reaction-induced changes in the sample surface's chemical composition are the cause of these impedance changes. The permeability of the magnetic material is modified as a result of this modulation of the chemical composition; this will be covered later in this study. Additionally, no change in impedance is seen when the potential decreases and the hydrogen evolution reaction (HER) is carried out, as shown in Fig. S3 (supporting information). It is evident that the impedance increases and decreases twice, respectively, after carrying out two oxidation and two reduction reactions. This provides additional evidence that the impedance change is caused by charge transfer in the redox reaction, which modifies the material’s permeability and is identified by impedance measurement.

**Figure 3:**
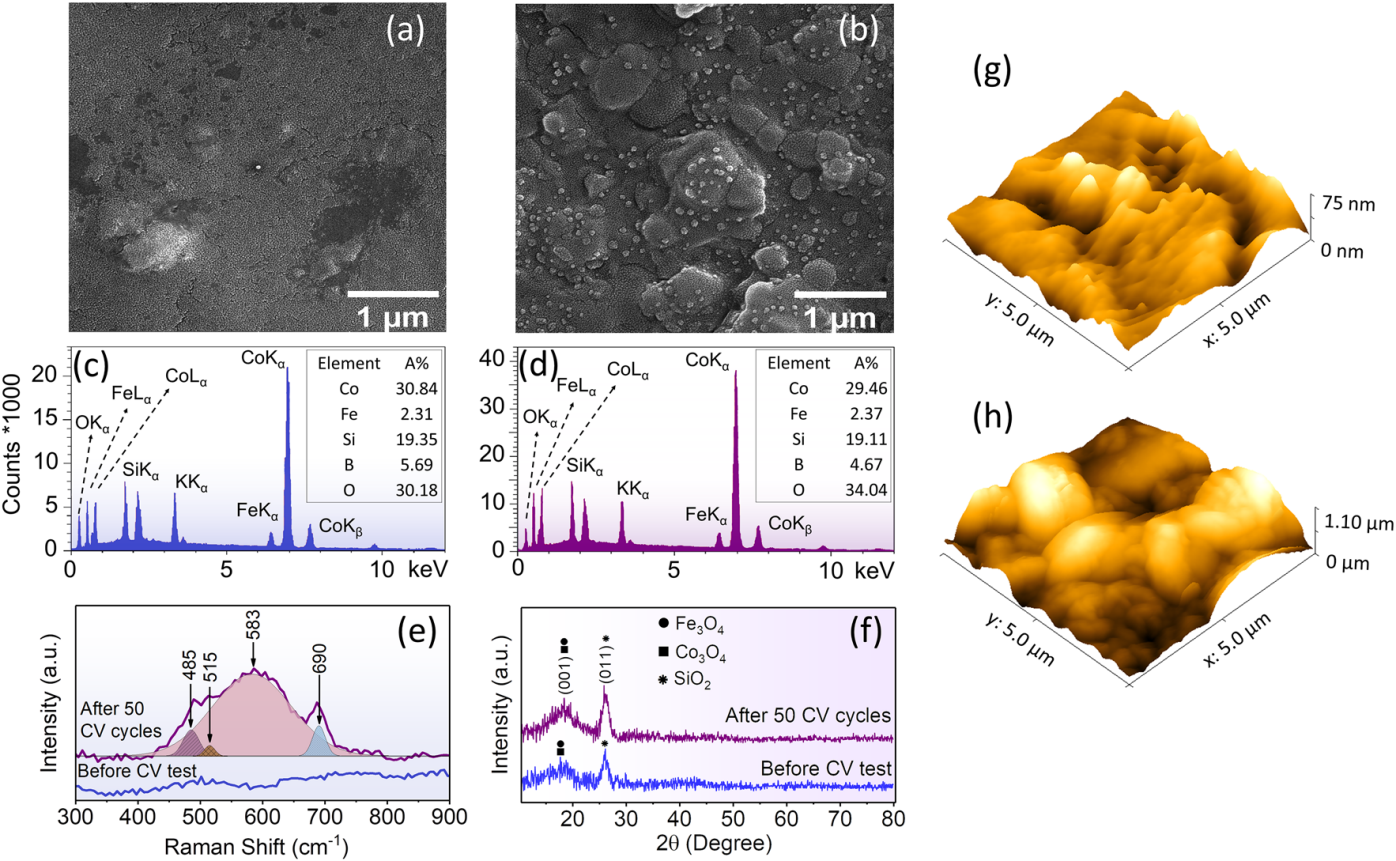
Field emission scanning electron microscope (FESEM) images of cobalt-based amorphous ribbon before and after 50 CV cycles (**a** and **b**), respectively. EDS spectrum of the sample before the CV test (**c**), and after the 50 CV cycles (**d**). Raman spectra of the cobalt-based ribbon before and after the CV test (**e**). Grazing incidence XRD (GI-XRD) results for the cobalt-based ribbon before and after the CV test (**f**). Atomic force microscope (AFM) images of the cobalt-based ribbon before and after the CV test (**g** and **h**).

In the following, it is focused on the structural changes at the sample surface due to irreversible manipulation after a couple of CV cycles. Field emission scanning electron microscope (FESEM) images of the pristine sample and the sample after 50 CV cycles have been prepared to investigate further the modifications in the surface structure of the sample, shown in Fig. 3a,b, respectively. By comparing the two FESEM images, it is clear that modifications have occurred in the sample surface after electrochemical reactions. It can be concluded that these modifications are the result of ionic interactions on the surface. Energy dispersive spectroscopy (EDS) characterization was performed on two samples to identify these species. Figure 3c,d show EDS characterization of the pristine and the sample after 50 CV cycles, respectively. By comparing the atomic ratio in the inset in Fig. 3c,d, we can see that the amount of oxygen increases after the redox reaction. Increasing the amount of oxygen can occur for two reasons. (i) After performing several electrochemical reactions, the sample surface is partially oxidized, and compounds of cobalt oxide and iron oxide are formed; (ii) By performing the oxidation reaction, ${OH}^{-}$ groups are bonded to the surface of the ribbon, which might not be eliminate after the reduction. We only see an increase in the oxygen element. It can be concluded that the species grown after the electrochemical reaction in Fig. 3b are related to cobalt oxide and iron oxide.

To identify the chemical structures at the sample surface, the Raman spectra have been prepared. Figure 3e shows the Raman spectra for the pristine cobalt-based amorphous ribbon and the sample after 50 CV cycles. Although the pristine sample has no peaks in the range of 300–900 cm^−1^, after performing an electrochemical reaction (50 CV cycles), four bands could be detected at 485, 515, 583, and 690 cm^−1^ with the help of fitting Raman data by Gaussian function. Three peaks at 485, 515, and 690 cm^−1^ are assigned to the spinel structure of Co_3_O_4_^32–37^. The transformation of CoO to the Co_3_O_4_ spinel structure has been studied^32,33^, and it has been reported that by changing the structure of cobalt oxide from cubic and hexagonal to spinel, these three characteristic peaks will appear in the Raman spectra. On the other hand, these peaks are not intense, which is due to the possibility of defects in the crystal structure of the spinel structure Co_3_O_4_. Ultimately, because of the partial oxidization of the sample surface, it is entirely expected that both CoO and Co_3_O_4_ exist at the surface. The redox reaction has been accomplished in the 1 molar KOH solution, so it is reasonable to assume some potassium-promoted spinal structures are performed. A broad peak at 583 cm^−1^ is in good agreement with the KCo_4_O_8_ Raman spectrum^38,39^. The results of an X-ray diffraction study (XRD) are displayed in Fig. 3f. Since the structure of the sample is amorphous, we do not expect intense peaks in the XRD spectrum. A broad peak at 18.2 degrees is related to Co_3_O_4_ in the Fd-3 m (ICSD-036256) space group and also to Fe_3_O_4_ in the Fd-3 m (ICSD-027898) space group. SiO_2_ is indexed in the P3121 (ICSD- 090,145) space group. XRD testing was unable to identify any crystalline structure at the sample surface due to numerous defects in the structure of the newly formed species and the amorphous nature of the sample. Finally, atomic force microscopy (AFM) analysis was conducted to determine the changing surface properties with electrochemical reactions. Three-dimensional (3D) AFM images of a 5 ⨯ 5 μm^2^ area are illustrated in Fig. 3g,h, respectively. From the AFM surface images, the average and RMS roughness for the sample before and after 50 cycles of electrochemical redox reaction are displayed in Table 1. Obviously, after completing 50 CV cycles of electrochemical redox reactions, new species grew on the sample surface and the roughness increased. Based on the previous discussion, it can be assumed that these are different cobalt oxide structures. While bonding ${OH}^{-}$ groups on the sample's surface modify the ferromagnetic amorphous ribbon's magnetic properties reversibly during each CV cycle, a percentage of cobalt oxide forms on the surface after each cycle ends, changing the magnetic characteristics irreversibly.

**Table 1:** Surface roughness analysis of the samples.

Sample	Mean roughness (nm)	RMS roughness (nm)
Before CV test	7.7	10.1
After 50 CV cycles	188.9	226.5

To demonstrate the surface dependence of the impedance changes we present two different experiments, presented in the following.

**Figure 4:**
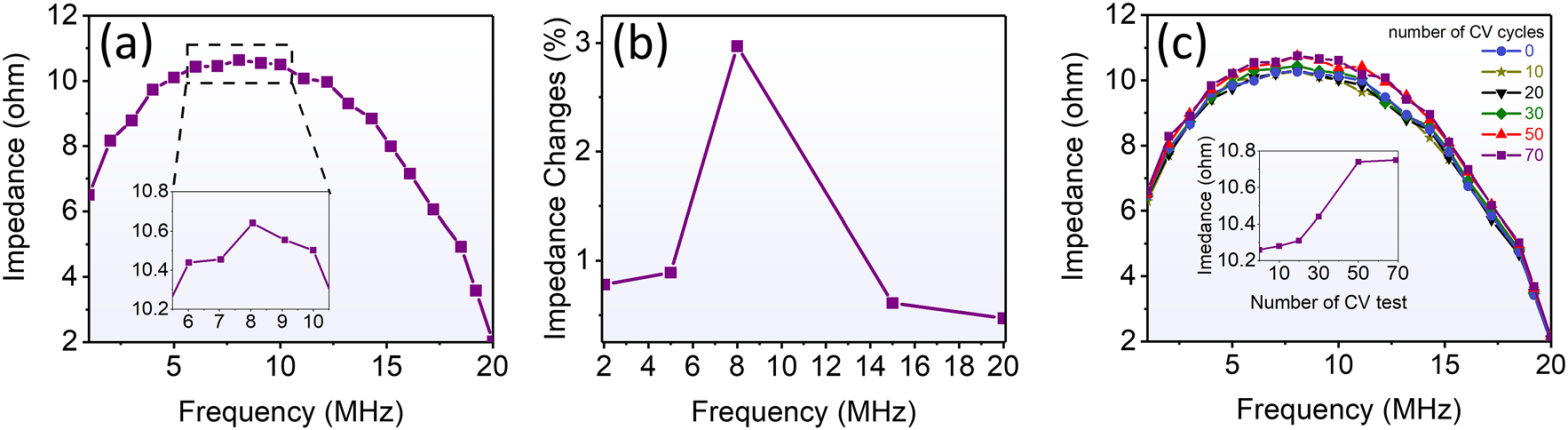
(**a**) Frequency sweep of impedance measurement. The maximum impedance and the lowest penetration depth appeared at the frequency of 8 MHz. (**b**) The impedance variation, which was caused by the redox reaction, vs frequency. (**c**) Irreversible increasing the impedance as the number of CV cycles (it remained unchanged after 50 CV cycles).

We have measured the impedance as a function of frequency $(f)$ to see how deep the electrochemical reaction can affect the magnetic properties of the CoFeSiB ribbon, Fig. 4a. To explain this behavior, first we note the impedance relation to the magnetic permeability. The impedance can be calculated by

1 $$Z={R}_{dc}jka{\text{coth}}(jka),$$

where ${R}_{dc}$ is the electrical resistance for a dc current, $j$ is the imaginary unit, $k=(1+j)/\delta$, and $2a$ is the thickness of the sample. And also, the skin depth is inversely related to the permeability and frequency, $\delta ={(\rho /\pi \mu f)}^{1/2}$, of metallic ferromagnet with electric resistivity $(\rho )$^40^. The impedance increases by increasing frequency up to 8 MHz and then declines by further increasing frequency. We should consider the relative contributions of domain wall motion and magnetization rotation in interpreting this effect^40^. The impedance reduction at high frequencies is due to the presence of eddy currents that cause damping of domain wall displacements; therefore, magnetic permeability declines and the impedance decreases. The CV test of the sample was carried out concurrently with the measurement of the impedance at frequencies of 2, 5, 8, 15, and 20 MHz in order to examine the relationship between electrochemical processes and impedance measurement. The experiment’s findings are shown in Fig. S4 (supporting information). A new parameter known as impedance changes has been defined as the difference between the maximum and minimum in the impedance curve in Fig. S4. Figure 4b shows this parameter as it has been researched as a function of frequency. This figure illustrates how the impedance changes reached their greatest value (about 3%) at 8 MHz. Based on Fig. 4a, it can be concluded that at this frequency, electrochemical processes have a detectable impact when the penetration depth reaches its lowest value (~ 100 nm)^41^. In fact, by performing electrochemical reactions at the surface of the sample, when a redox reaction occurs, charge transporting between the ionic agents (${OH}^{-}$) in the electrolyte and the metallic elements at the sample surface (for example, cobalt) reversibly affects the permeability of the magnetic material, which is detectable just at the frequency of 8 MHz. This directly results from the surface-limited nature of changes in chemical compounds caused by electrochemical processes, which is made apparent by the concentrated current at the surface. Impedance evolutions are therefore only apparent at this frequency.

Additionally, as was previously mentioned, when an electrochemical reaction is conducted at the surface of the cobalt-based amorphous ribbon, surface chemistry is modified both reversibly and irreversibly. In irreversible manipulation, after each CV cycle, the surface of the sample is partially oxidized, and finally, after 50 CV cycles, cobalt oxide and iron oxide are formed at the surface. These new species change the impedance since not only their resistance but also their permeability are different. In fact, cobalt oxide and iron oxide have poor electrical conductivity; hence, the impedance increases due to a decrease in conductivity at the sample surface irreversibly. The result of this measurement is shown in Fig. 4c. The impedance has been measured as a function of frequency at different CV cycle numbers. In the inset of Fig. 4c the maximum impedance, which occurred at the frequency of 8 MHz, is depicted versus the number CV test. It can be seen that the impedance increased as a function of CV cycles and reached a stable state after 50 CV cycles.

**Figure 5:**
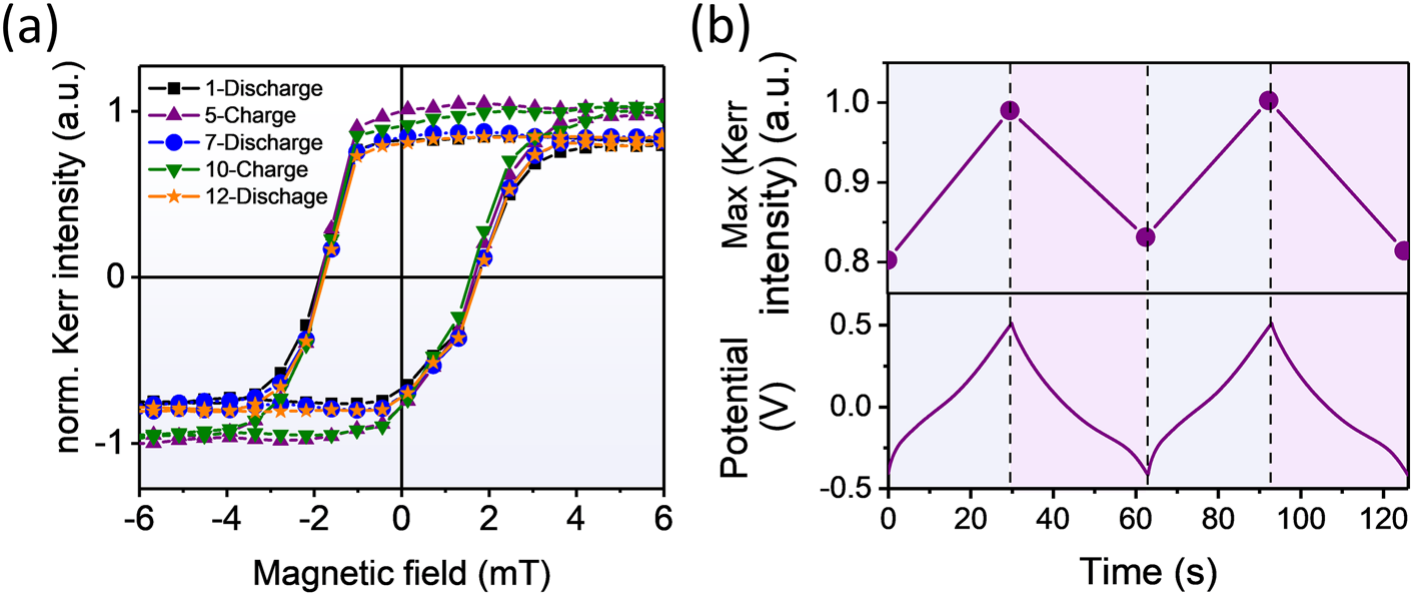
Redox reaction dependence of magnetic properties of cobalt-based amorphous ribbon. (**a**) In-plan magnetization curve for two charge and discharge states. (**b**) Fully- reversible variation in magnetic response of the sample with respect to the charge–discharge cycles under a constant current of 100 μA.

The other surface characterization experiment we performed was the MOKE. We obtained two separate oxidation and reduction states at the sample's surface by charging it with a continuous current of 100 μA at two different voltage polarities (− 0.4 V and 0.5 V). Figure 5a shows the MOKE result in charge and discharge modes. With each charge of the sample, we see an increase in the Kerr intensity; by discharging, the Kerr intensity returns to the previous state. Charging and discharging reactions are entirely reversible, so it can be concluded that this magneto-optical characterization is tightly correlated to the reversible manipulation of the cobalt-based amorphous ribbon’s surface by redox reaction. Figure 5b shows a diagram of changes in the Kerr intensity and charge discharge simultaneously.

## Conclusions

We have demonstrated that impedance spectroscopy can be an instant analysis method to study modifications to the MI phenomenon. In addition, we manipulated the magnetic properties of a cobalt-based thick layer by an electrochemical redox reaction in both fully reversible and irreversible ways, which was investigated by impedance measurement. During a CV cycle by oxidation of the sample the impedance increased reversibly by about 0.2 Ω and decreased reversibly by about 0.1 Ω by reduction. After completing each CV cycle, the surface of the sample remained partially oxidized, so that after 50 CV cycles, it reached a stable state. By increasing the CV cycles beyond about 50, the system gets stable state, and the impedance shows a reversible behavior despite the oxidation and reduction in the electrochemical cell. Our results open a new path to developing the characterization mechanisms in MI phenomena studying.

## Methods

The conventional melt-spinning method was used to fabricate a Co-based amorphous alloy (Co_60_Fe_3_Si_12_B_25_) with dimensions of 0.8 mm width, 20 μm thickness, and 52 mm length. All electrochemical measurements were performed in a three-electrode cell. The counter and reference electrodes were platinum and Ag/AgCl, respectively. All analyses were measured in 1 M KOH. Surface morphology and composition of the samples were analyzed by field emission scanning electron microscopy (FESEM, MIRA3) equipped with an energy dispersive spectrometer (EDS). The four-point probe method was used to measure the impedance. For the impedance measurements an alternating AC voltage, produced by the function generator (GPS-2125), passes through the sample’s length and a 50 Ohm resistor in series with the sample. The impedance is measured by measuring the potential of the sample sides using an oscilloscope (GPS-1102B). A setup was designed to conduct the impedance and electrochemical measurements simultaneously. The details of this setup are described in Fig. S1 (Supporting information). Raman spectroscopy was conducted at room temperature with a Teksan Raman microscope spectrometer (Takram P50C0R10, λ = 532 nm). X-ray diffraction (XRD) measurements were performed using a Philips PW1730 diffractometer with Cu Kα1 radiation (λ = 1.5405 Å). The topography of the sample surfaces was imaged by a non-contact mode (EasyScan 2, Nanosurf) Atomic Force Microscope (AFM) under ambient conditions.

### Supplementary Information

Supplementary Information.

## Data Availability

The related data are available from the corresponding author on request.
