# Supplementary material for: Skin-effect-mediated magnetoionic control of charge transport in thick layers
Source: Sci Rep. 2024 Feb 9;14:3332. doi: 10.1038/s41598-024-53970-9 (PMC10858034; doi:10.1038/s41598-024-53970-9)
Supplement: Supplementary file 1 — Supplementary Information. [file 41598_2024_53970_MOESM1_ESM.docx]

**Supporting information for: Skin-effect-mediated magnetoionic control of charge transport in thick layers**

The diagram of the experimental setup is visible in Figure S1. To conduct the electrochemical measurements, a conventional three-electrode electrochemical cell was employed, and the impedance of the cobalt-based amorphous ribbon (Co_60_Fe_3_Si_12_B_25_) was measured concurrently. The skin depth, which is approximately 100 nanometers, is apparent in this figure.

The raw data and smoothed curve of the impedance changes during the oxidation and reduction process at the sample's surface are depicted in Figure S2. Due to not only the limitations of the equipment for this project but also the lower magnitude of impedance changes (about 2 Ω) rather than the sample impedance (10.7 Ω), the impact of noise became non-negligible, hence using the smoothing process was unavoidable. Based on the results, it can be inferred that the impedance undergoes an increase and decrease of approximately 2 and 1 ohm, respectively, during the oxidation and reduction reactions.

Figure S3 illustrates that while the impedance changes due to oxidation or reduction at the sample's surface, it remains unaffected by an increase in current density at the hydrogen evolution reaction region. This observation provides evidence that the impedance is solely influenced by electrochemical reactions occurring at the surface of the sample.

To demonstrate the surface limitation of the magnetoionic process, impedance measurements were taken at various frequencies, as shown in Figure S4. The results indicate that the most changes in the impedance occurs at the frequency of 8 MHz, which corresponds to the smallest value of the skin depth. This finding suggests that a concentrated current at the surface is capable of detecting surface-limited changes in chemical compounds.


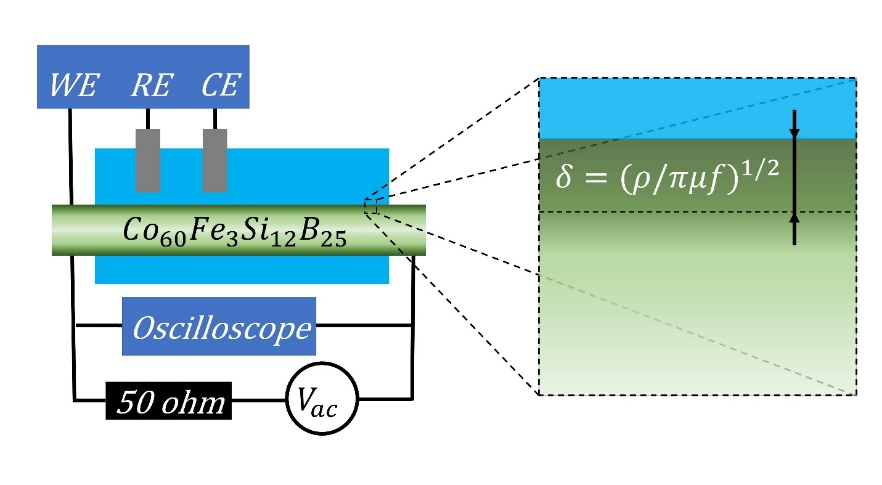


Figure S1: The schematic of the setup. WE: working electrode, RE: reference electrode, CE: counter electrode.


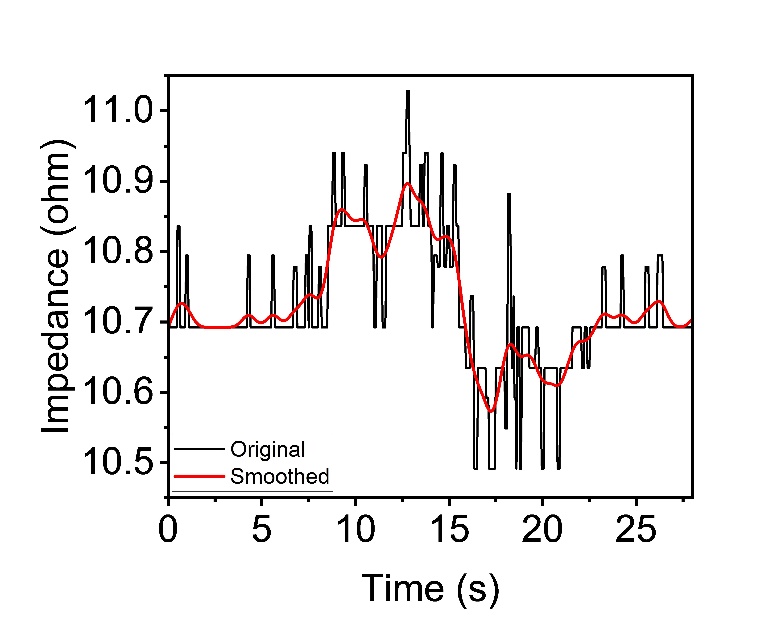


Figure S2: Raw data and smoothed curve of impedance changes in two oxidation and reduction states.


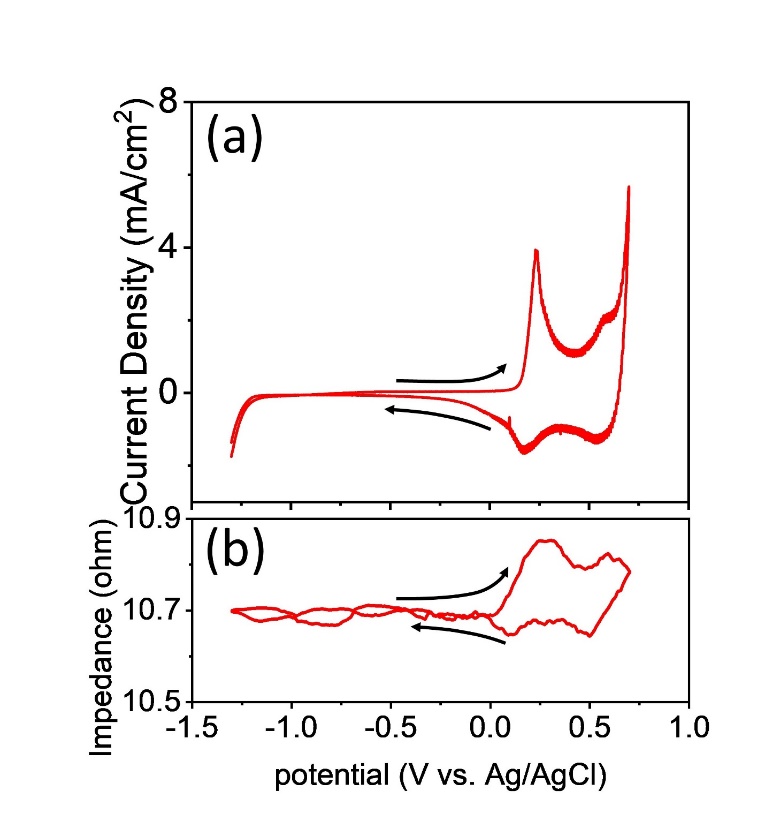


Figure S3: (a): Cyclic voltammetry test of the Co-based ribbon. (b): Impedance measurement at the frequency of 8 MHz, simultaneously with the CV test.


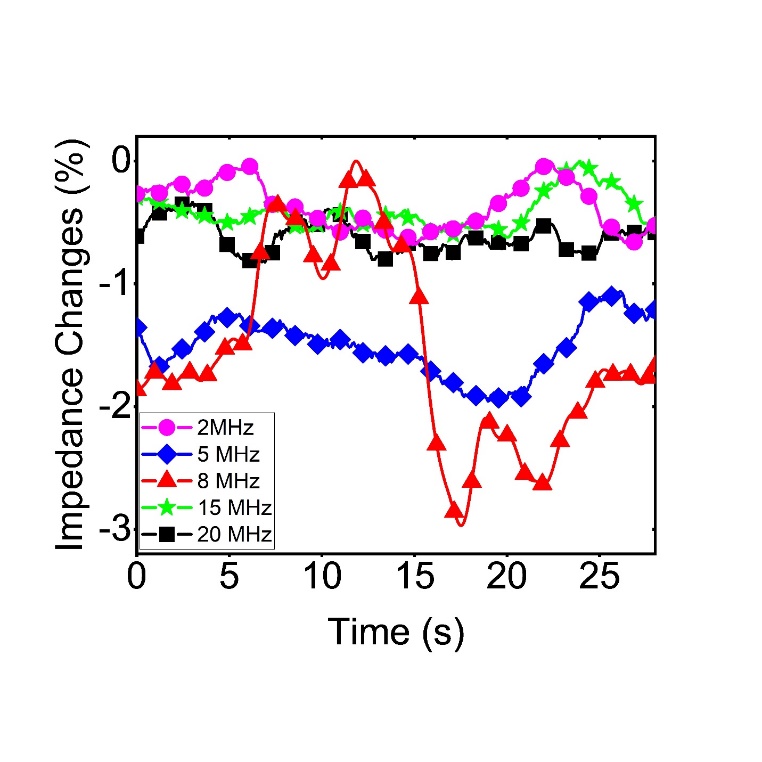


Figure S4: The impedance measurement at two oxidation and reduction states which has been done at different frequencies.
